# Degradation of Polymethylmethacrylate (PMMA) Bioreactors Used for Algal Cultivation

**DOI:** 10.3390/ma16134873

**Published:** 2023-07-07

**Authors:** Ewa Borucinska, Przemyslaw Zamojski, Wojciech Grodzki, Urszula Blaszczak, Izabela Zglobicka, Marcin Zielinski, Krzysztof J. Kurzydlowski

**Affiliations:** 1 Faculty of Mechanical Engineering, Bialystok University of Technology, 15-351 Bialystok, Poland; p.zamojski@pb.edu.pl (P.Z.); w.grodzki@pb.edu.pl (W.G.); i.zglobicka@pb.edu.pl (I.Z.); k.kurzydlowski@pb.edu.pl (K.J.K.); 2 Faculty of Electrical Engineering, Bialystok University of Technology, 15-351 Bialystok, Poland; u.blaszczak@pb.edu.pl; 3 Faculty of Geoengineering, University of Warmia and Mazury in Olsztyn, 10-720 Olsztyn, Poland; chudy@uwm.edu.pl

**Keywords:** algae, bioreactors, polymethylmethacrylate, characterization, mechanical properties, transmission, material properties

## Abstract

This paper depicts characteristics of degradation of walls of bioreactors made of polymethylmethacrylate (PMMA) which was used to culture algae. The degradation processes take place stimulated by lighting of external surface and interaction with cultured species on internal surface. Results presented are representative for degradation of a bioreactor tube after the 4-year cultivation of *Chlorella* sp. Microscopic observations, roughness and transmission tests showed that changes have occurred on the inner surface. The result of use is a decrease in transmission and an increase in roughness. Microscopic observations showed that particles remained after culture, especially in cracks.

## 1. Introduction

Bioreactors are systems that support an active biological environment [1] by providing suitable, stable conditions for cell growth and metabolism with control of operating parameters. This equipment should provide constant conditions for cultured microorganisms, such as food and gas supply, lighting (in case of photobioreactors), maintaining constant temperature and pH [2].

Photobioreactors (PBRs) are systems that enable the cultivation of photoautotrophic organisms with the usage of dedicated light sources (sunlight or artificial) [3]. PBRs can be divided into open (natural waters—lakes, lagoons, ponds; artificial ponds and containers) and closed PBRs (flat-plate, column, tubular PBRs) [4]. The open pond has several limitations, including poor productivity, requirement for an outdoor area, restriction to certain microalgae strains, poor light utilization as well as constant water evaporation [5,6]. Closed PBRs are made with walls transparent to light, like glass or some plastics. The main disadvantage of such reactors is gradual degradation of the light transmitting walls, among others because of deposition biofilm on the inner surface. Although these bioreactors are more expensive than open ones [7,8], they are widely used in industry [9] for production of biofuels [7], biomass [10], animal feed supplements [11,12], flue gas and wastewater treatment [13,14]. It should be also noted that algae cultures absorb CO_2_ during space exploration missions [11,15].

Criteria used in the selection of PBRs are efficiency, reliability and process stability [16,17]. Due to the differences in the requirements of various cultured organisms, it is difficult to design universal bioreactor [17]. In particular, one needs to take into account required light and supply of culture gases or food [18]. In the context of light penetration, material of choice for reactor walls are transparent plastics such as Plexiglas, polycarbonate, acrylic, polyvinyl chloride (PVC) and polyethylene (PE). Glass is less often used because of its inherent brittleness [9] and lower capacity for shaping.

Reactor walls made of polymeric materials are prone to degradation because of processes taking place under the influence of light, culture solution, flowing gas pumped and biofilm formation [4]. Light transmitted to the reactor is also causing a breakdown of the macromolecules of polymeric material of the wall (photodegradation) [4].

Generally, it is assumed that products made of PMMA degrade because of exposure to light radiation, temperature and water [19,20,21,22]. Temperature-induced degradation can be neglected in the case of bioreactors for cultivation algae, which operate at nearly constant temperature and under negligible temperature gradient across the PMMA wall. To light irradiation is exposed the external surface of the reactor walls. The inner wall is in contact with microorganisms and flowing medium, in some cases a two phase (liquid + gas) of variable chemistry. Thus, there are distinct degradation factors acting on the outer and inner surfaces of reactors used for cultivation of algae.

It should be noted that one of the consequences of reactor wall degradation is an increase in surface roughness. Increased surface roughness affects light transmission and stimulates growth of biofilm further accelerating reduction of light transmission to the tube [4,7,15,23]. In this paper, we adopted an experimental approach to the walls degradation by conducting investigations of a reactor used continuously for algal cultivation over the period of 4 years.

## 2. Materials and Methods

The bioreactor investigated was used for 1460 days. During this period, at least 60–80 culture cycles were carried out. The breeding cycle lasted about 7 days. The reactor wall was made of PMMA. The PMMA tube of the reactor has a 75 mm outer diameter and a wall thickness of 2 mm. The capacity of the tube was 2 L. Synthetic medium N-BBM+V (Bold Basal Medium with three-fold nitrogen and vitamins; modified) with pH ranging from 6.4 to 6.8 was used to grow *Chlorella* sp. algae. In Aquael Plant 2600 Lumen (AQUAEL sp. zo.o., Suwalki, Poland), 8000 K, (24 h/24 h) lighting was used. The bioreactor was operated at room temperature (18–22 °C), with no additional cooling or heating of the tube. After every seven days of cultivation, reactor was emptied. During the cleaning procedure, reactor was filled with a 1.5% solution of amino sulfonic acid and aired for 12 h. Then, the reactor was emptied and triple times flushed with water, twice using tap water and the last one with distilled water. During flushing, reactor was filled with water for a half hour and aired.

**Figure 1:**
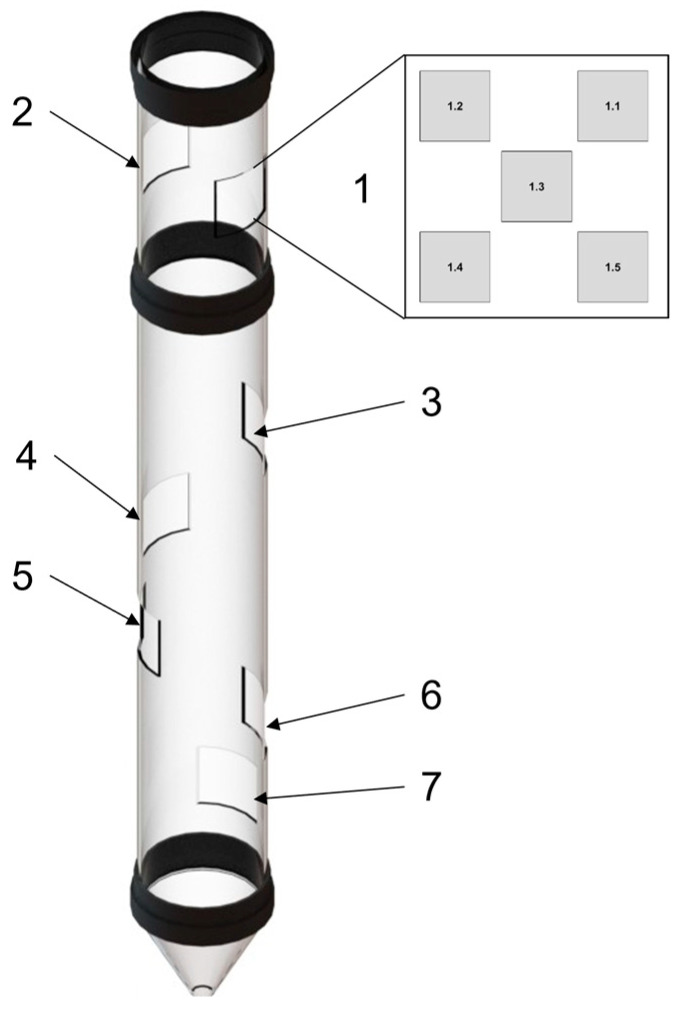
Location and designation of samples cut-out of the reactor tube.

In order to investigate the degree and mode of degradation of PMMA tube, samples were cut-out from seven characteristic sections as shown in Figure 1. Each sample was divided into five sub-areas of 5 mm × 5 mm (Figure 1), which were subjected to microscopic observations (inner surface of the tube) and spectral transmission testing (incident light on inner surface). As a reference material, a commercially available PMMA was used, which is referred to as 0 sample.

Observations of the wall surface were conducted using VHX-7000 (KEYENCE, Osaka, Japan) and Scanning Electron Microscope Scios2 (Scios2 DualBeam, Thermo Fisher Scientific, Waltham, MA, USA) using acceleration voltage of 2 kV. The samples before observation on Scanning Electron Microscope were coated with a 7 nm layer of gold. 

Spectral transmission characteristics of the samples have been determined using spectrometer Stellarnet (StellarNet, Inc., Tampa, FL, USA). Measurements have been conducted in five test—observation areas. Results have been compared to the data for a reference sample of virgin PMMA tube. Measurements of the light transmission were performed in a dark room. A tungsten halogen lamp with fiberoptic output and the fiberoptic spectrometer was used. Illumination was perpendicular to the surface. Light transmittance was determined with light source placed against outer side of the wall with the detector positioned against the inner side. 

Roughness of the samples was determined with Hommel tester t1000 (Hommelwerke GmbH, Waltrop, Germany). Parameters were measured at the sample locations marked on Figure 1. The measurements were performed using the contact method. Surface topography of the PMMA was investigated on inner the inner side exposed to the contact with the medium. The device probe was moving with a speed of 0.5 mm/s. The ISO 11562(M1) [24] filter was used to filter the signal. The parameters evaluated were R_a_ (arithmetic average of profile height deviations from the mean line) and R_z_ (maximum peak to valley height of the profile, within a single sampling length) [25]. 

Static three-point bending tests were carried out on seven specimens using an MTS Insight (MTS Systems Corporation, Eden Prairie, MN, USA) device with DIC System Aramis 4M software (GOM, Braunschweig, Germany). No pre-tension was applied. The tests were carried out until sample disintegration.

## 3. Results and Discussion

**Figure 2:**
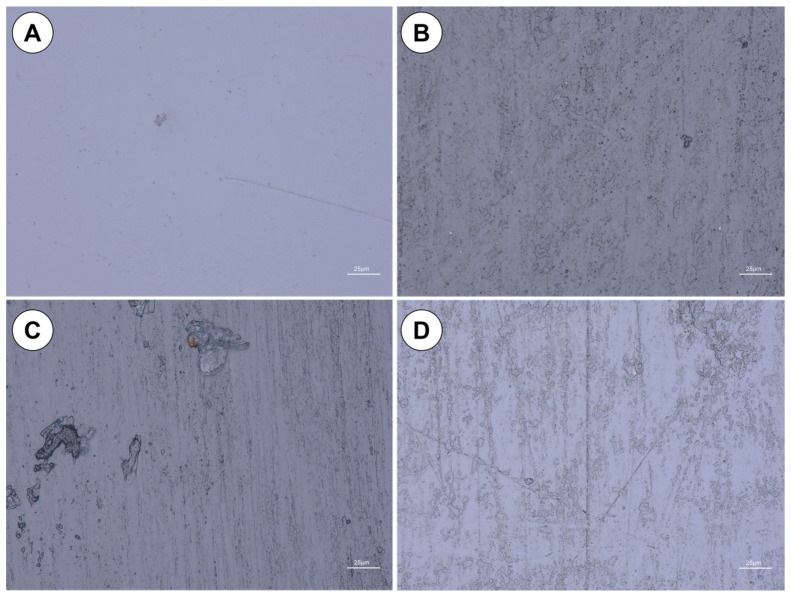
Light microscope images of inner wall surface: (**A**) Reference sample; (**B**) Sample 3; (**C**) Sample 5; (**D**) Sample 7.

Characteristic images of the inner surface of cylinder wall are shown in Figure 2. The images reveal traces of wear in form of scratches distributed over the entire tube surface. The scratches are mainly horizontal, with very few oblique/vertical scratches.

Regarding processes leading to formation of the vertical scratches on the inner wall of reactors, we suggest that of major importance is vertical flow along the walls induced by the circulation of the medium, which is a suspension of cultivated algae. The abrasive nature may be exhibited by undissolved particles of mineral salts used in the culture medium [26] and algae themselves. Since the bioreactor under study was tested only post-mortem, without testing during in-service time, no information was available on the rate of degradation. Systematic studies of that rate are in progress.

Using an image analysis program, number of scratches per intersection line was counted from micrographs taken at magnification of approximately 1500×. The results of scratch counting are given in Table 1. It can be noted from the data in Table 1 that number of scratches varies from 1 per 6.75 × 104 μm^2^ for the reference sample to 48 for sample 3 from the middle part of the tube. It should be also noted that smaller number of scratches appear in the upper part of the tube. This may suggest that the scratches might be caused by flow of particles accumulated in the reactor because of biological activity of algae culture.

**Figure 3:**
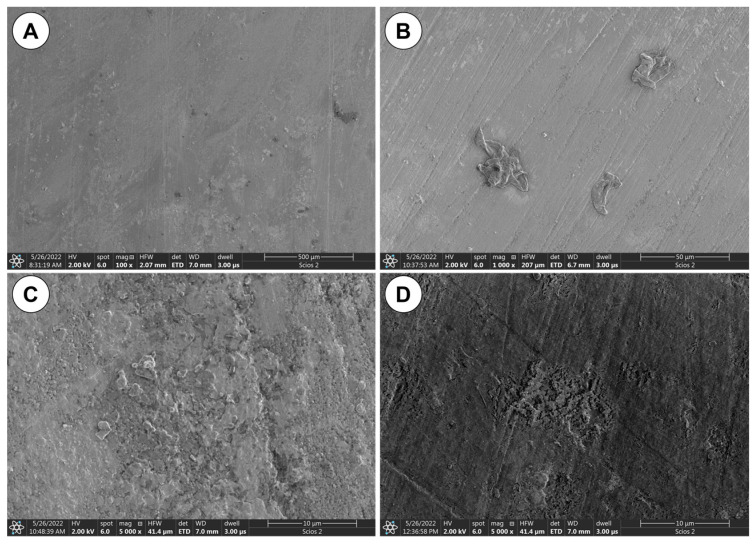
SEM images of tube wall inner surface: (**A**) Sample 1; (**B**) Sample 3; (**C**) Sample 4; (**D**) Sample 7.

Scanning electron microscopy (SEM) combined with elemental analysis (EDS) revealed that the particles attached to inner surface most likely are biological residues from algae culture—see Figure 3. It can be argued that mechanical damage to the surface of the bioreactor tube becomes a preferable site for adhesion of organic particles.

Light-induced degradation of PMMA has been subject of many papers—see for example [19,20,22,27,28,29,30] and references included there. It has been shown that degradation is particularly intensive under exposure to UV irradiation and depends on the dose [19,22,27,28,30]. UV-curing process of the PMMA occurs due to the crosslinking reaction between the ester side chains of two neighboring PMMA polymer molecules even at an excimer wavelength below 250 nm. At a medium irradiation dose, side chain cleavage from the polymer main chain takes place yielding mechanical densification of the polymeric material due to Van der Waals forces with a subsequent increase of the refractive index. At a high irradiation dose, polymer main chain scission occurs, resulting in total defragmentation of the polymer structure. The result of the described changes can be observed by examining the transmission of light through the sample.

**Figure 4:**
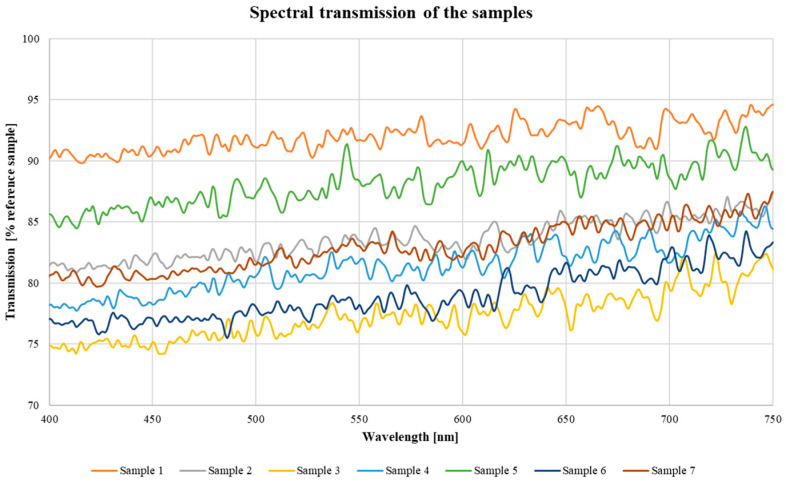
Spectral light transmission characteristics of bioreactor samples.

Results of the measurements of spectral transmission are shown in Figure 4 as a percentage of the values obtained for the reference sample. It can be noted that light transmission is reduced along the entire surface of the bioreactor. The transmission reduction varies from 5 to 25% depending on wavelength and location along the reactor tube. Higher light attenuation has been measured for the wavelength in the range 400–450 nm, i.e., in the blue range of photosynthesis. This finding clearly indicates substantial consequences of the degradation process of the bioreactor wall to the efficiency of algae growth.

The changes in transmission do not occur uniformly and are dependent on the location of the sample on the bioreactor. The samples with the lowest transmission values (sample 3 and sample 6) are located next to each other vertically. The low transmission of these samples may indicate the similar conditions that prevailed around this section of the tube. The sample with the highest transmission relative to the reference sample was taken from the top of the tube, which may indicate that it was not in constant contact with the culture medium.

**Figure 5:**
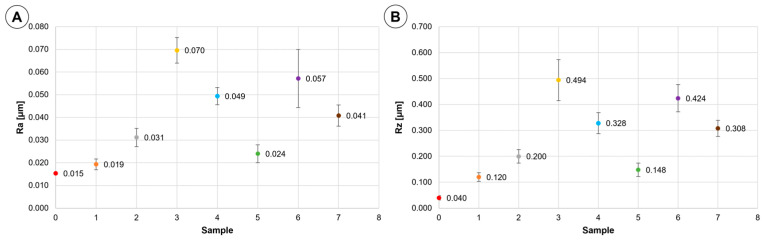
The results of roughness measurements: (**A**) R_a_ values; (**B**) R_z_ values. Designation of the samples: ● Sample 0; ● Sample 1; ● Sample 2; ● Sample 3; ● Sample 4; ● Sample 5; ● Sample 6; ● Sample 7.

Results of roughness measurements, R_a_ and R_z_ parameters, are shown in Figure 5. It can be noted that the roughness parameters used in this study are significantly increased with respect to virgin material. In a sense, this is another manifestation of changes in topography of the tube inner surface, which is addressed already in the section of scratch density.

**Figure 6:**
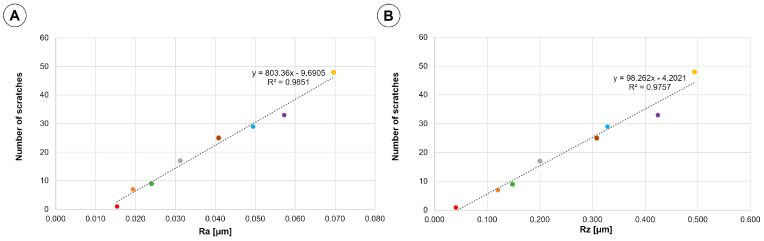
The results of number of scratches measurements plotted against the roughness measurements: (**A**) R_a_ values; (**B**) R_z_ values. Designation of the samples: ● Sample 0; ● Sample 1; ● Sample 2; ● Sample 3; ● Sample 4; ● Sample 5; ● Sample 6; ● Sample 7.

In fact, it can be concluded that roughness values correlate with the number of scratches as shown in Figure 6, which presents scratch density plotted against R_a_ and R_z_. Thus, we may conclude that the scratches are major features of topography of the inner surface of the bioreactor.

Density of scratches plotted against R_a_ (a) and R_z_ (b) values is presented in Figure 6. The dependence of the number of cracks on roughness is close to linear as evidenced by an R^2^ value of 0.9851 for R_a_ and 0.9757 for R_z_.

**Figure 7:**
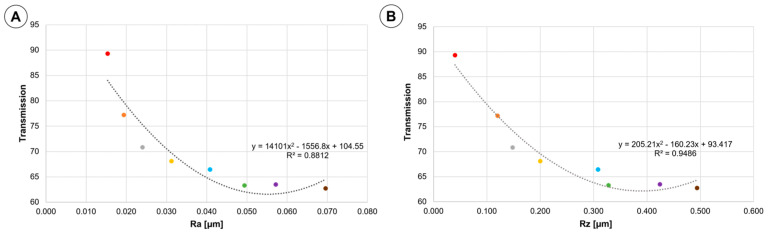
The results of light transmission for wave length 450 nm measurements plotted against the roughness measurements: (**A**) R_a_ values; (**B**) R_z_ values. Designation of the samples: ● Sample 0; ● Sample 1; ● Sample 2; ● Sample 3; ● Sample 4; ● Sample 5; ● Sample 6; ● Sample 7.

In order to elucidate the effect of changes in topography on light transmission, the results from Figure 4 are plotted against values of R_a_ and R_z_ in Figure 7. A monotonic decrease in light transmission is observed with increasing roughness parameters in agreement with theory of light attenuation [31]. Changes in the transmission value are related to the roughness value by a polynomial relationship. The fit of the data to the graph—R^2^ is 0.8812 for the R_a_ parameter, while for the R_z_ parameter 0.9486. Surface roughness effect on transparency of PMMA has been reported in [20,29]. This phenomenon has also clear theoretical explanation in the context of surface topography effect on light reflected and transmitted by transparent elements. Among others, aligned and regular scratches on the surface, revealed in the present study, are expected to influence transmitted light, giving rational to our conjecture [20,29].

Static three-point bending tests were used to estimate apparent Young modulus. The obtained values are listed in Table 2. It can be concluded that the average value of apparent Young modulus of 3.0 GPa agrees with the literature data [32] and is not affected by degradation processes. This implies that degradation phenomena are taking place in a relatively narrow zones near to the inner and outer wall surface.

Fracture surface in specimens subjected to a three-point bending reveals the feature typical of cracking in a brittle manner, which is consistent with the literature data [29,30].

Results of a long-time exposure to solar (UV) and artificial (UV) lamp radiations typical of bioreactors have been reported in [21]. As in some other studies, degradation of PMMA was primarily monitored by measuring changes in the mechanical properties. It has been found that that exposure to solar (UV) radiation induced higher degradation than that induced by artificial. It should be noted, however, that generally degradation of mechanical properties of irradiated specimens is relatively mild and do not impart brittleness of PMMA [21].

Similar conclusions have been drawn regarding the degradation effect of exposure to water [21]. A long-time exposure to water, sea and fresh, results in mass gain in the range 1.5 to 2.0%, bringing about reduction of stiffness and tensile strength, however, not to a critical level from the point of view of remaining in-service life of bioreactors. In fact, this is a key point of the present study that among various consequences of in-service degradation of PMMA walls of bioreactors for growing algae, loss of transparency, as evidenced by our results, overweighs reduction in the mechanical properties. Further, we put forward a conjecture that the observed loss of transparency is related to the increase of roughness of inner surface of PMMA walls. An experimental basis for this is polynomial correlation between surface roughness and transparency demonstrated in Figure 7.

## 4. Conclusions

The results obtained in the present study show that degradation of the PMMA wall tube is primarily manifested by changes in topography of the inner surface. This degradation reduces significantly light transmission (450 nm), particularly in the middle part of the vertical tube. The inner surface degradation is manifested by vertical scratches and deposition of biological debris. No degradation has been found in mechanical strength of the wall material. There is a valid value of R_a_ of 0.025 μm above which transmission falls significantly. In case of R_z_, the same has been observed above the value of 0.15 μm.

The relationship between light transmission and roughness of the wall tube can be used for in-service estimates of wall transparency with no need for removing content of bioreactors.

## Figures and Tables

**Table 1:** Measured density of scratches per 6.75 × 10^4^ μm^2^.

Sample	0	1	2	3	4	5	6	7
**Number of scratches per 6.75 × 10^4^ μm^2^**	1	7	17	48	29	9	33	25

**Table 2:** Value of apparent Young modulus as determined in a three-point bending tests.

Sample	1	2	3	4	5	6	7	Average
**Young Modulus [GPa]**	3.03	2.92	3.00	3.08	2.92	2.90	2.86	3.00

## Data Availability

All data generated or analyzed during this study are included in this published article.
